# Determinants of work and social participation in patients with psoriatic arthritis in the Netherlands: an observational study

**DOI:** 10.1186/s41927-022-00279-7

**Published:** 2022-08-17

**Authors:** Tamara W. van Hal, Michelle L. M. Mulder, Mark H. Wenink, Johanna E. Vriezekolk

**Affiliations:** 1 Department of Rheumatology, Sint Maartenskliniek, P.O. Box 9011, 6500 GM Nijmegen, The Netherlands; 2 Radboud Institute for Health Sciences (RIHS), Radboud University Medical Center, Nijmegen, The Netherlands; 3 Department of Research, Sint Maartenskliniek, Nijmegen, The Netherlands

**Keywords:** Psoriatic arthritis, Work outcomes, Absenteeism, Presenteeism, Unemployment

## Abstract

**Background:**

Psoriatic arthritis can cause pain, disability, and permanent joint damage. This can lead to impairments in work and social participation. Little is known about the extent of these impairments in routine practice. With this study, we aim to examine the extent of work and activity impairment in (subgroups of) Dutch patients with psoriatic arthritis (PsA), and to examine determinants associated with this impairment.

**Methods:**

This is an observational study using data collected from the electronic health records of PsA patients treated at the Sint Maartenskliniek, the Netherlands. Data about work and activity impairment were collected via the Work Productivity and Activity Impairment questionnaire. To compare our PsA-cohort with the Dutch general population, we used age- and sex-matched data derived from the Central Bureau of Statistics. Regression analyses were performed to examine determinants of work and activity impairment.

**Results:**

In total, 246 patients were included, of which 126 (51.2%) were female. Mean age (S.D.) was 55.7 (13.2) years. Compared with the Dutch general population, work for pay (WFP) was significantly lower in PsA (52.9% versus 62.6%, *P* < 0.001). In PsA, younger age and better physical function were associated with WFP status (*P* < 0.05). Higher disease activity, worse physical function, and worse mental health-related quality of life were associated with both more work and activity impairment (*P* < 0.05). Furthermore, reaching low disease activity status (LDA) according to Psoriatic ArthritiS Disease Activity Score (PASDAS; ≤ 3.2) was associated with less work and activity impairment than reaching LDA according to DAS28-CRP (≤ 2.9) (*P* < 0.05).

**Conclusions:**

In PsA patients, worse physical function was associated with a lower likelihood of having WFP, and higher work and activity impairment. PASDAS LDA as a goal for treat to target, compared to DAS28-CRP, appears to favour the reduction of work and activity impairment.

**Supplementary Information:**

The online version contains supplementary material available at 10.1186/s41927-022-00279-7.

## Background

Psoriatic arthritis (PsA) is an immune-mediated inflammatory disease of joints and entheses, which can lead to pain, disability, and a loss of quality of life (QoL) [1]. All these may culminate in impairments in work, leading to a loss in employment and productivity. PsA patients are less likely to have work-for-pay (WFP) than healthy controls [2]. Even when having WFP, the work impairment caused by PsA is reported to be between 24 and 38 percent of total potential work productivity [3, 4]. PsA may also lead to impairment in social activities, which can have a direct impact on social relations, intimacy, and community participation [5]. When identifying which areas of impairment are most important to patients, the “activities and participation” domain is mentioned most often [6]. To thoroughly assess the impact of disease on daily life, for example with the aim of evaluating whether a treatment is cost-effective, a better understanding of the extent of work and social participation and its influencing factors is vital.

Work and social participation are influenced by both disease-related and societal factors. In spondyloarthritis (SpA), a multinational study showed differences in work participation and work impairment between countries, which can be partly explained by economic factors (e.g., health care expenditure), or by cultural differences (e.g., perceived importance of employment) [7].

The effect of disease-related factors on work participation is exemplified by the fact that higher levels of disease activity and disability have been associated with an increase in work impairment, while in clinical trials treatment of active disease led to a decrease in both work and social impairment [8–10]. However, the differences in setting (clinical trial versus real world, international versus national) make it hard to extrapolate international data to other countries and patient populations. A valid estimation of the societal impact of a disease is, however, crucial when allocating resources for treatment.

The aim of this study was to examine the employment status of PsA patients in a Dutch routine practice cohort, compared with an age- and sex-matched Dutch general population. We also examined the associations of work impairment and activity impairment with patient and disease characteristics. Finally, we examined the association of low disease activity (LDA) status, measured by PsA-specific Psoriatic Arthritis Disease Activity Score (PASDAS) and Disease Activity Score of 28 joints (DAS28-CRP), with work and activity impairment.

## Material and methods

### Aim of the study

To examine the extent of work and activity impairment in (subgroups of) patients with PsA, and to examine determinants associated with this impairment.

### Study design and population

This study describes the baseline data of a longitudinal study, conducted at the department of Rheumatology in the Sint Maartenskliniek in Nijmegen, the Netherlands. Patients with rheumatologist-diagnosed PsA, aged ≥ 18 years, were eligible for this study. Patients were treated according to local protocol, which is based on PASDAS driven treat-to-target (T2T) from March 2019 onwards. Before March 2019, patients were treated according to a DAS28-CRP based protocol [11]. We approached patients by sending them a questionnaire about work and activity impairment at the moment they were switching from the DAS28-CRP to the PASDAS driven strategy. Only the clinical data of those patients who returned the questionnaire were gathered for further analysis. Data was collected between July 2019 and December 2020.

### Data collection

The Work Productivity Activity Impairment: Specific Health Problem (WPAI-SHP) questionnaire was used to collect data about work and activity impairment [12]. With the WPAI-SHP, patient’s WFP status, absenteeism (percentage of the time being away from work due to the specific health problem) and presenteeism (percentage of productivity loss while at work due to the specific health problem) and activity impairment (percentage of “productivity” loss during non-work activities due to the specific health problem) are assessed. The work parameters can be combined to estimate overall work impairment as follows:

$$\left( {{\text{Absenteeism }} + \, \left( {\left( { \, 1 \, {-}{\text{ absenteeism}}} \right) \, *{\text{ presenteeism}}} \right)} \right)$$

The electronic health record of patients with PsA was used to extract data about demographics, treatment, disease activity, functional impairment, and health-related QoL (HR-QoL). Disease activity was measured via DAS28-CRP and/or PASDAS [13]. The PASDAS is a PsA-specific composite disease activity score that consists of a 68 tender joint count, a 66 swollen joint count, a six entheses Leeds enthesitis index (LEI) [14], a twenty digit dactylitis count, and a C-reactive protein (CRP). These are complemented with a visual analogue scale (VAS) of global disease activity by both patient and physician (range: 0–100 mm) and the physical summary component score (PCS) of the Short Form 12 (SF-12; range: 0–100). Next to the PCS, the SF-12 also yields a mental summary component score (MCS; range: 0–100) [15]. A higher score in PASDAS defines a higher disease activity. Cut-off points for near-remission and LDA state are 1.9 and 3.2, respectively [16].

To strengthen our analysis of the effect of LDA status on work and activity impairment, we used both the PsA-specific PASDAS and the DAS28-CRP. While this latter composite disease activity score was originally developed for use in rheumatoid arthritis (RA), and despite the fact that a 28 joint based score is not advised for PsA [17], the DAS28-CRP is still often used for PsA [18, 19]. A higher score in DAS28-CRP defines a higher disease activity. We used the cut-off points as defined for RA: 2.4 for remission and 2.9 for LDA, respectively [20].

Physical impairment was measured routinely with the Health Assessment Questionnaire-Disability Index (HAQ). This questionnaire evaluates physical disability in eight different domains (dressing/grooming, arising, eating, walking, hygiene, reach, grip, and activities). Scores range from zero (no impairment at all) to three (unable to perform a certain task). Although originally developed for RA, the HAQ-DI has been validated for PsA [21].

### Statistical analysis

Continuous data were described with the mean (S.D.) or median (with interquartile ranges, IQR), when appropriate. Categorical data were described as absolute frequencies and percentages.

To compare our PsA-cohort with the Dutch general population, we used an age- and sex-matched model based on data from the Central Bureau of Statistics (CBS) of the Netherlands [22]. The CBS provided data on WFP status, stratified for sex and age groups (per five years of age). Differences between our PsA-cohort and the general population were tested using a Chi square test.

Using complete cases only, the relationship between WFP and demographics, disease activity, functional impairment, mental component HR-QoL, and therapy modality (no systemic treatment, convential systemic DMARD (csDMARD) or biological/targeted DMARD (b/tsDMARD)) was investigated with a logistic regression model. WFP status (yes/no) was the dependent variable. The relationships of overall work impairment and activity impairment with demographics, disease activity, functional impairment, HR-QoL, and therapy modality were tested with a linear regression model. Overall work impairment or activity impairment were the dependent variables. After univariable regression analyses, independent variables with a *P* < 0.157 (Akaike criterion) were entered in a multivariable regression model using backward stepwise selection.

Differences with respect to WFP/overall work impairment/activity impairment between groups of different disease activity states (i.e. remission/LDA) were tested using Chi square or Mann–Whitney U.

As a sensitivity analysis, we created a dataset where missing data were imputed using multiple imputation with chained equations (MICE); 54 complete data sets were iterated [23]. Imputed variables included: WFP, overall work impairment, activity impairment, absenteeism, presenteeism, PASDAS, DAS28-CRP, PCS, MCS, 28/68 tender joint count, 28/66 swollen joint count, LEI, dactylitis count, patient global VAS, physician global VAS, CRP, and HAQ.

All statistical procedures were carried out in STATA v13.0 (StataCorp, USA).

## Results

### Response rate and patient characteristics

Four hundred sixty patients were approached for this study; 264 patients filled out the WPAI questionnaire (response rate 53.5%). Of these 246 patients, 162 (65.9%) had a valid PASDAS score, 173 (68.1%) had a valid SF-12 score, and 113 (45.9%) had a valid HAQ-DI.

Table 1 shows the demographic and disease characteristics of the study population. Fifty-one percent of the participants was female and mean age was 55.7 years (S.D. 13.2 years). Hundred and sixteen patients (47.5%) used csDMARD only, 94 (38.2%) used b/tsDMARD (with or without csDMARD), whereas 36 patients (14.6%) used no systemic treatment. Mean PASDAS was 3.04 (S.D. 1.40); 54% of patients were in PASDAS LDA (≤ 3.2). Mean DAS28-CRP was 2.17 (S.D. 0.93); 80% were in DAS28CRP LDA (≤ 2.9).

**Table 1:** Patient and disease characteristics of PART2-cohort

Age (years)		55.7 (13.2)
Female—N (%)		126 (51.2%)
PASDAS^a^	Mean	3.04 (1.40)
	LDA (≤ 3.2)–N (%)	87 (53.7%)
	Remission (≤ 1.9)–N(%)	37 (22.7%)
DAS28-CRP	Mean	2.17 (0.93)
	LDA (2.9)–N (%)	183 (79.9%)
	Remission (2.4)–N (%)	159 (69.4%)
SJC68—N (%)	0	169 (68.7%)
	1–4	60 (24.4%)
	5 or more	12 (5.3%)
TJC68–N (%)	0	117 (47.6%)
	1–4	79 (32.1%)
	5 or more	45 (18.3%)
LEI–N (%)	0	185 (75.2%)
	1	22 (8.9%)
	2 or more	22 (8.9%)
Active dactylitis–N (%)		5 (2.0%)
Global VAS	Physician	14.4 (15.3)
	Patient	31.6 (23.5)
CRP		3.76 (8.6)
SF12	PCS^b^	41.6 (10.2)
	MCS^b^	49.1 (10.6)
HAQ^c^		0.63 (0.6)
DMARD use–N (%)	None	36 (14.6%)
	csDMARD	116 (47.5%)
	b/tsDMARD	94 (38.2%)

All in mean (SD), unless stated otherwise. Variables with > 10% missing are marked

*b/ts DMARD* biological/targeted systemic DMARD, *CRP *C-reactive protein, *csDMARD* conventional systemic DMARD, *DAS28-CRP*, disease activity score of 28 joints using CRP, *DMARD *disease modifying antirheumatic drug, *HAQ *health assessment questionnaire disability index, *MCS *mental summary component score, *PASDAS *psoriatic arthritis disease activity score, *PCS *physical summary component score, *SF12 *short form-12, *SJC66 *swollen joint count of 68 joints, *TJC68 *tender joint count of 66 joints, *VAS *visual analog scale

^a^PASDAS was known in 162 patients

^b^SF12 was known in 173 patients

^c^HAQ was known in 113 patients

### Work for pay, overall work impairment, and activity impairment

Table 2 shows the WFP status and degree of overall work impairment and activity impairment in our PsA-cohort. 52.9% of the patients with PsA (N = 130) had WFP, compared to 62.6% in the age- and sex-matched model of the general population (*P* < 0.001). In patients who had WFP, median absenteeism, presenteeism, and overall work impairment were 0% (IQR 0%–0%), 20% (IQR 0%–40%), and 10% (IQR 0%–40%), respectively. Activity impairment for the whole sample (N = 246) was 30% (IQR 10%–60%).

**Table 2:** Percentage employment and impairment in PART2-cohort

Work for pay–N (%)		130 (52.9%)
Absenteeism	when working	0% (0%–0%)
Presenteeism	when working	20% (0%–40%)
Overall work impairment	when working	10% (0%–40%)
Activity impairment	all participants	30% (10%–60%)

All in median (IQR), unless stated otherwise

### Associations between work/activity impairment and patient/disease characteristics

Table 3 shows the results of both univariable and multivariable regression analyses, of the associations between WFP/impairment, and both patient and disease characteristics.

**Table 3:** Determinants associated with work for pay, overall work impairment and activity impairment

	Work for pay (n = 90)		Overall work impairment (n = 49)			Activity impairment (n = 92)		
	Univariable (OR)	Multivariable (OR)	Univariable	Multivariable	Standardized	Univariable	Multivariable	Standardized
Age	0.91* (0.89, 0.94)	0.89* (0.84, 0.94)	− 0.16 (− 0.62, 0.30)			0.10 (− 0.17, 0.36)		
Female sex	0.86 (0.52, 1.42)		16.06* (6.09, 26.03)			12.82* (6.11, 19.53)		
PASDAS	(0.44, 0.75)		15.75* (12.02, 19.47)	6.65* (1.39, 11.91)	0.32*	14.33* (12.12, 16.54)	6.82* (3.32, 10.32)	0.35*
MCS	1.02 (0.99, 1.05)		− 1.38* (− 1.93, -0.82)	− 0.65* (− 1.27,− 0.03)	− 0.24*	− 1.27* (− 1.63, -0.91)	− 0.45* (− 0.86,− 0.03)	− 0.17*
HAQ	0.32* (0.17, 0.63)	0.22* (0.08, 0.55)	25.11* (15.20, 35.02)	19.95* (10.31, 29.59)	0.46*	25.82* (19.49, 32.14)	18.17* (11.66, 24.69)	0.45*
No DMARD	0.73 (0.34, 1.56)		2.59 (− 13.48, 18.66)			6.29 (− 3.55, − 16.12)		
b/tsDMARD	1.12 (0.65, 1.95)		− 0.03 (− 10.58, 10.52)			− 0.65 (− 7.75, 6.45)		

Associations between work for pay and independent variables were studied using logistic regression. Associations between overall work impairment/activity impairment and independent variables were studied using linear regression. Number of patients included in the multivariable model is shown above the table. Regression coefficients with 95% confidence intervals are shown, unless stated otherwise

*b/tsDMARD *biological/targeted synthetic DMARD, *DMARD *disease modifying anti rheumatic drug, *HAQ* health assessment questionnaire disability index, *MCS *mental summary component score, *PASDAS *psoriatic arthritis disease activity score

^*^P =  < 0.05

#### Work for pay

Univariable logistic regression analyses showed significant associations between a positive WFP status and younger age (OR = 0.91, *P* < 0.001), lower PASDAS (OR = 0.57, *P* < 0.001), and lower HAQ scores (OR = 0.32, *P* < 0.001). In the multivariable model, only age (OR = 0.89, *P* < 0.001) and HAQ (OR = 0.22, *P* = 0.001) remained significant, explaining 34% of the variance.

#### Overall work impairment

Univariable linear regression analyses showed significant associations between higher overall work impairment and female sex (*B* = 16.1, *P* = 0.002), higher PASDAS (*B* = 15.7, *P* < 0.001), lower MCS (*B* =− 1.4, *P* < 0.001), and higher HAQ (*B* = 25.1, *P* < 0.001). In the multivariable model, the associations between overall work impairment and PASDAS (β = 0.32, *P* = 0.014), HAQ (β = 0.46, *P* < 0.001), and MCS (β = -0.24, *P* = 0.04) remained significant, explaining 61% of the variance.

#### Activity impairment

Univariable linear regression analyses showed significant associations between higher activity impairment and female sex (*B* = 12.8, *P* < 0.001), higher PASDAS (*B* = 14.3, *P* < 0.001), lower MCS (*B* = − 1.3, *P* < 0.001), and higher HAQ (*B* = 25.8, *P* < 0.001). In the multivariable model, the associations between activity impairment and PASDAS (β = 0.35, *P* < 0.001), MCS (β = − 0.17, *P* = 0.03), and HAQ (β = 0.45, *P* < 0.001) remained significant, explaining 61% of the variance.

#### Sensitivity analyses with imputed data set

Additional file 1: Table S1 shows the results of univariable and multivariable regression analyses using the imputed data set. These results are in line with the complete case analyses, with the following differences. For WFP, in the multivariable model, HAQ was no longer associated with WFP. Instead, a lower PASDAS showed a significant relationship with a positive WFP status (OR = 0.59, *P* < 0.001). Moreover, the multivariable model showed an additional significant association of a higher activity impairment with female sex (*B* = *5.2*, *P* = 0.04).

### Differences in work and activity impairment between patients in low disease activity according to either PASDAS or DAS28-CRP

Additional file 1: Table S2 shows the number and frequency of patients by disease activity status (LDA or remission). Of the 163 patients with valid PASDAS scores, 129 (79%) were in LDA according to DAS28-CRP (≤ 2.9), and 88 (54%) were in LDA according to PASDAS (≤ 3.2). Forty three patients (26%) were in LDA according to DAS28-CRP, but not according to PASDAS.

**Fig. 1:**
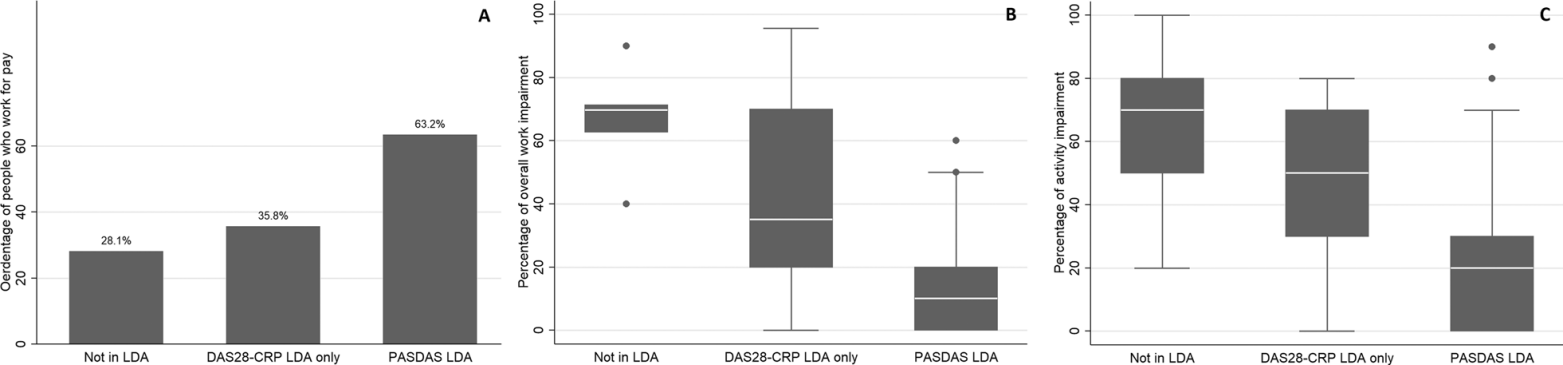
Work for pay, overall work impairment and activity impairment by disease activity status. **A** Work for pay. **B** Work impairment. **C** Activity impairment. Work for pay in N (%). Overall work impairment and activity impairment in median (IQR). Overall work impairment only described in patient who have work for pay. CRP = C-reactive protein; DAS28-CRP = Disease Activity Score of 28 joints using CRP; PASDAS = Psoriatic ArthritiS Disease Activity Score

Table 4 and Fig. 1 show WFP, overall work and activity impairment of patients in LDA according to either PASDAS or DAS28-CRP. Subgroup analyses between patients in PASDAS LDA (N = 88) and patients in DAS28-CRP LDA (N = 129) showed that patients in PASDAS LDA were more likely to have WFP than patients in DAS28-CRP LDA (respectively 63% and 54%). In patients who had WFP, median overall work impairment did not differ between the patients in PASDAS LDA or DAS28-CRP LDA (i.e. 10% in both groups). Median activity impairment was lower in patients in PASDAS LDA compared to patients in DAS28-CRP LDA (20% versus 30%).

**Table 4:** Proportion of patients with work for pay, work impairment and activity impairment by low disease activity and remission status

	Low disease activity			(Near) Remission		
	DAS28-CRP (all) N = 127	DAS28-CRP (not in PASDAS) N = 42	PASDAS N = 87	DAS28-CRP (all) N = 108	DAS28-CRP (not in PASDAS) N = 71	PASDAS N = 37
Work for pay	106 (54.3%)	15* (35.8%)	55 (63.2%)	61 (56.5%)	36 (50.7%)	25 (67.6%)
Overall work impairment	10% (0%, 30%)	35%* (20%, 70%)	10% (0%, 20%)	10% (0%, 30%)	20%* (10, 40%)	0% (0%, 10%)
Activity impairment	30% (10%, 50%)	50%* (30%, 70%)	20% (0%, 30%)	20% (0%, 50%)	30%* (20%, 60%)	0% (0%, 20%)

Work for pay is expressed in N (%). Overall work impairment and activity impairment are expressed in median (IQR). Outcomes of patient in DAS28-CRP LDA/remission (but not in PASDAS LDA/remission) were tested against outcomes of patients in PASDAS LDA/remission using either Chi-square or Mann Whitney U

*CRP *C-reactive protein, *DAS28-CRP *disease activity score of 28 joints using CRP, *LDA *low disease activity, *PASDAS* psoriatic arthritis disease activity score

^*^P =  < 0.05

Further subgroup analyses showed that patients who were in DAS28-CRP LDA, but not in PASDAS LDA (N = 43), were less likely to have WFP than patients who were also in PASDAS LDA (N = 86): 34% versus 63%, *P* = 0.02. Patients who were in DAS28-CRP LDA, but not in PASDAS LDA, showed more overall work impairment (20% versus 0%, *P* < 0.001) and more activity impairment (30% versus 0%, *P* < 0.01) than patients who were also in PASDAS LDA.

Table 4 shows WFP, overall work impairment, and activity impairment of patients in (near)-remission according to either PASDAS or DAS28-CRP. Comparable results were found.

## Discussion

In this cross-sectional study, we explored the impact of PsA on work and social activities and examined determinants associated with work and activity impairment. We found a significant lower employment rate (WFP) in PsA patients compared to an age- and sex-matched Dutch general population. Furthermore, we found that older age and a worse physical function were related to poorer WFP status. Overall work impairment and activity impairment both were related to higher disease activity, worse physical function and worse mental health status. Lastly, we found that being in PASDAS LDA (compared to DAS28-CRP LDA) increased the likelihood of having WFP, and was associated with better work-related outcomes.

Around 53% of the patients with PsA in our cohort had WFP; this corresponds with the lower bound of the employment rates found in several systematic reviews [24–26]. While the included patients in these latter reviews came from North America, South America, and Europe, no Dutch patients were included. Also, in these reviews there was a predominance of clinical centers from the United States and Canada. International differences in both the accessibility of health care as well as provision of unemployment benefits could account for the lower amount of patients with WFP in our cohort. Dutch employers are obliged to provide paid sick leave for up to two years, after which there is a possibility to apply for social disability benefits. An absence of paid sick leave or social disability benefits could urge employees to keep working while sick. Also, the Dutch sociopolitical system provides access to reimbursed healthcare via mandatory health care insurance. With this insurance, a wide range of effective DMARD’s is accessible to all citizens. This access to effective treatments may lead to better disease control, and therefore to less loss of work force or less work impairment. Noteworthy, the employment rates found in our PsA-cohort was also lower than another Dutch cohort of patients with early PsA (mean symptom duration 1.0 years, employment rate 74%)[27]. Given that our routine practice cohort comprises PsA patients with various disease duration, this suggests that a longer disease duration could negatively affect the likelihood of having WFP. Unfortunately, we were not able to explore the relationship between disease duration and WPF, as disease duration data were not available for our study.

In both multivariable models, over 60% of work and activity impairment was explained by the combined effects of higher disease activity, worse mental HR-QoL, and worse physical function. This suggests that these determinants are highly relevant factors to decrease the societal burden of PsA. First, with respect to disease activity, the association between work impairment and a higher disease activity in this routine care cohort is in line with the results of previous clinical trials. When compared with placebo, treatment with tumor necrosis alpha inhibitors or ixekizumab either improved work productivity or lowered overall work impairment [10, 28, 29]. This would even support a causal relationship between disease activity and work impairment. However, in contrast to previous studies, we did not find an association between therapy modality and work impairment [27, 30]. In contrast to our study, the study of Tillett et al. [30] showed a large difference in disease activity between patients who were treated with csDMARD or bDMARD. Given that disease activity was related to work and activity impairment in our cohort, while therapy modality was not, this may indicate that a stringent disease control is key to preventing impairment (either in work or non-work activities), regardless of the way this disease control is achieved.

Second, a worse mental HR-QoL was robustly associated with both work and activity impairment. To our knowledge, this association has not been reported before. With this current design, we cannot infer a causal relationship between mental well-being and impairment. Given that mental HR-QoL remained significant in the multivariable model together with disease activity and physical function, this indicates that the relationship is independent of disease activity and functional impairment. Longitudinal and interventional data are needed to determine the directionality of the relationship between mental HR-QoL and work and social impairment.

Third, a worse physical function was also associated with both work and activity impairment. This finding is consistent with a study of Tillett et al. [10]. In fact, this study even used the HAQ as an anchor to find the minimal clinical important difference of the WPAI:SPH in PsA. While our analyses cannot infer a causal relationship, it is tempting to speculate that a worse physical function leads to more impairment in both work and non-work activities.

Last, we found that being in PASDAS LDA (compared to DAS28-CRP LDA) increases the likelihood of having WFP, and is associated with lower overall work impairment and activity impairment. We previously reported that the PsA-specific PASDAS revealed residual inflammation when compared to the DAS28-CRP [31]. In line with these findings, we observed more WFP and less work and activity impairment when employing the LDA criteria of the PASDAS instead of the DAS28-CRP. All these results may indicate that T2T using PsA-specific targets may lead to better disease control, and thus less impairment.

A major strength of our study is the study setting. The PsA-cohort of the Sint Maartenskliniek is a real world cohort, which facilitates extrapolation of our results to real world cohorts in other out-patient clinics. Our cohort is treated following a PASDAS-driven T2T strategy, which entails that on every visit we collect data about disease activity and QoL [11]. However, this real world outpatient setting (in contrast to a dedicated study setting such as a randomized controlled trial) also means that parameters not essential to the primary treatment goal may be missing more often.

One limitation of our study was indeed a substantial amount of missing data, mostly regarding the SF-12 or the HAQ questionnaires. To examine whether this may have led to biased results, we conducted sensitivity analyses with an imputed data set. For WFP, the multivariable analysis using the original data set with only complete cases showed a significant association between having WFP and a higher HAQ, but not with PASDAS. The multivariable analysis using the imputed data set showed a significant association between having WFP and a lower PASDAS, but not with HAQ. In our opinion, there is an interplay between WFP on the one hand and disease activity/physical function on the other hand. Our study design, however, limits inferences about the directionality of these relationships.

Regarding activity impairment, the imputed multivariable model showed an additional association with female sex. Earlier research by our group showed significant differences between men and women in disease activity scores [32]. Further research is needed to explore whether the association between activity impairment and female sex is a true association or a spurious relationship, when in reality the differences in activity impairment are related to the differences in disease activity between the sexes.

Another limitation is the possibility of responder bias. Privacy regulations limited us in gathering data about the non-responders. However, we compared the patient and disease characteristics of our responders with previously published data about the PsA cohort in our clinic [31]. Our subset of this population showed a slight overrepresentation of women (51% in our study versus 46% in the study of Mulder et al.), but comparable PASDAS and HAQ scores, and use of DMARD’S, making it conceivable that our results are valid.

Taking together, our study findings imply that PsA has an impact on those aspects of life that patients hold most dearly [6]. We showed robust relationships between work and activity impairment, and disease activity. Also, we showed that reaching LDA by definition of the PsA-specific PASDAS (in comparison to the widely-used, but not PsA-specific DAS28-CRP) is associated with a higher likelihood of being employed, and less work and activity impairment. Therefore, it is conceivable that stringent T2T with a PsA-specific disease activity score may improve patients’ ability to perform both work and non-work activities. Supported by the results of Wervers et al. [27], we suggest that early achievement of LDA may prevent loss of employment.

## Conclusions

Our study revealed that approximately 53% of patients in our routine practice PsA-cohort were employed. Higher disease activity, worse physical function, and mental wellbeing independently contributed to work and activity impairment. Furthermore, patients with a PASDAS LDA status reported less impairment in work and social activities than patients with a DAS28-CRP LDA status. Whether a T2T approach with a PsA-specific disease activity score has a positive effect on work and activity impairment remains to be investigated.

## Supplementary Information

**Additional file1: Table S1**. Determinants associated with work for pay, overall work impairment and activity impairment using the imputed data set**. Table S2. **Patients split by LDA and remission status.

## Data Availability

The data underlying this article will be shared on reasonable request to the corresponding author.

## Abbreviations

b/tsDMARD: Biological/targeted synthetic DMARD
CBS: Central Bureau of Statistics
CRP: C-reactive protein
csDMARD: conventional synthetic DMARD
DAS: Disease Activity Score
DMARD: Disease Modifying AntiRheumatic Drug
HAQ: Health Assessment Questionnaire–disability index
HR-QoL: Health-Related QoL
IQR: InterQuartile Range
LDA: Low Disease Activity
LEI: Leeds Enthesitis Index
MCS: Mental summary Component Score
MICE: Multiple Imputation with Chained Equations
OR: Odds Ratio
PASDAS: Psoriatic ArthritiS Disease Activity Score
PCS: Physical summary Component Score
PsA: Psoriatic Arthritis
QoL: Quality of Life
RA: Rheumatoid Arthritis
S.D.: Standard Deviation
SF12: Short Form 12
SHP: Specific Health Problem
SpA: SpondyloArthritis
T2T: Treat-to-Target
VAS: Visual Analogue Scale
WFP: Work For Pay
WPAI: Work Productivity and Activity Impairment
