# Supplementary material for: Determinants of work and social participation in patients with psoriatic arthritis in the Netherlands: an observational study
Source: BMC Rheumatol. 2022 Aug 17;6:49. doi: 10.1186/s41927-022-00279-7 (PMC9382787; doi:10.1186/s41927-022-00279-7)
Supplement: Supplementary file 1 — Additional file1: Table S1. Determinants associated with work for pay, overall work impairment and activity impairment using the imputed data set. Table S2. Patients split by LDA and remission status. [file 41927_2022_279_MOESM1_ESM.docx]

**Supplementary table 1:** **Determinants associated with work for pay, overall work impairment and activity impairment using the imputed data set**

|  | Work for pay (n=243) | | Overall work impairment (n=130) | | Activity impairment (n=246) | |
| --- | --- | --- | --- | --- | --- | --- |
|  | Univariable (OR) | Multivariable (OR) | Univariable | Multivariable | Univariable | Multivariable |
| Age | 0.91  (0.89, 0.94) | 0.91*  (0.89, 0.94) | -0.19  (-0.67, 0.28) |  | 0.10  (-0.16, 0.37) |  |
| Female sex | 0.85  (0.52, 1.42) |  | 17.31*  (6.94, 27.68) |  | 12.92*  (6.22, 19.62) | 5.12*  (0.10, 10.14) |
| PASDAS | 0.62*  (0.50, 0.77) | 0.59*  (0.46, 0.75) | 15.56*  (11.78, 19.34) | 8.36*  (3.54, 13.18) | 13.61*  (11.59, 15.63) | 8.07*  (5.53, 10.61) |
| MCS | 1.01  (0.98, 1.04) |  | -1.40*  (-1.95, -0.86) | - 0.86*  (-1.39, -0.34) | -1.14*  (-1.48, -0.80) | -0.59*  (-0.91, -0.26) |
| HAQ | 0.48*  (0.29, 0.80) |  | 25.72*  (16.36, 35.07) | 15.50*  (6.16, 24.84) | 23.69*  (17.91, 29.48) | 14.15*  (8.93, 19.37) |
| No DMARD | 0.69  (0.34, 1.42) |  | 2.26  (-14.26, 18.78) |  | 6.65  (-3.09, 16.40) |  |
| bDMARD | 1.21  (0.72, 2.03) |  | -1.82  (-12.83, 9.19) |  | -0.77  (-7.86, 6.32) |  |

Associations between work for pay and independent variables were studied using logistic regression. Associations between overall work impairment/activity impairment and independent variables were studied using linear regression. Number of patients included in the multivariable model is shown above the table. Regression coefficients with 95% confidence intervals are shown.

b/tsDMARD = biological / targeted synthetic DMARD; DMARD = Disease Modifying Anti Rheumatic Drug; HAQ = Health Assessment Questionnaire Disability Index; MCS = Mental summary Component Score; PASDAS = Psoriatic ArthritiS Disease Activity Score

* *P* = < 0.05

**Supplementary table 2: patients split by LDA and remission status**

|  | | PASDAS LDA | |
| --- | --- | --- | --- |
|  |  | No | Yes |
| DAS28-CRP LDA | No | 32  (19.6%) | 2  (1.2%) |
|  | Yes | 43  (26.4%) | 86  (52.8%) |

|  | | PASDAS Near-remission | |
| --- | --- | --- | --- |
|  |  | No | Yes |
| DAS28-CRP  Remission | No | 53  (32.5%) | 0  (0%) |
|  | Yes | 72  (44.2%) | 38  (23.3%) |

CRP = C-reactive protein; DAS28-CRP = Disease Activity Score of 28 joints using CRP; LDA = Low Disease Activity; PASDAS = Psoriatic ArthritiS Disease Activity Score
